# New Methods for Assessing the Fascinating Nature of Nature Experiences

**DOI:** 10.1371/journal.pone.0065332

**Published:** 2013-07-26

**Authors:** Yannick Joye, Roos Pals, Linda Steg, Ben Lewis Evans

**Affiliations:** 1 Research Center for Marketing and Consumer Science – Research Foundation Flanders (FWO), Faculty of Business and Economics, University of Leuven, Leuven, Belgium; 2 School of Social Studies, Hanze University of Applied Sciences, Groningen, The Netherlands; 3 Department of Psychology, Faculty of Behavioural and Social Sciences, University of Groningen, Groningen, The Netherlands; Université Pierre et Marie Curie, France

## Abstract

In recent years, numerous environmental psychology studies have demonstrated that contact with nature as opposed to urban settings can improve an individual’s mood, can lead to increased levels of vitality, and can offer an opportunity to recover from stress. According to Attention Restoration Theory (ART) the restorative potential of natural environments is situated in the fact that nature can replenish depleted attentional resources. This replenishment takes place, in part, because nature is deemed to be a source of fascination, with fascination being described as having an “attentional”, an “affective” and an “effort” dimension. However, the claim that fascination with nature involves these three dimensions is to a large extent based on intuition or derived from introspection-based measurement methods, such as self-reports. In three studies, we aimed to more objectively assess whether these three dimensions indeed applied to experiences related to natural environments, before any (attentional) depletion has taken place. The instruments that were used were: (a) the affect misattribution procedure (Study 1), (b) the dot probe paradigm (Study 2) and (c) a cognitively effortful task (Study 3). These instrument were respectively aimed at verifying the affective, attentional and effort dimension of fascination. Overall, the results provide objective evidence for the claims made within the ART framework, that natural as opposed to urban settings are affectively positive (cfr., affective dimension) and that people have an attentional bias to natural (rather than urban) environments (cfr., attentional dimension). The results regarding the effort dimension are less straightforward, and suggest that this dimension only becomes important in sufficiently difficult cognitive tasks.

## Introduction

*“Workin’ 9 to 5, what a way to make a livin’.*

*Barely gettin’ by, it’s all takin’ and no givin’.*

*They just use your mind and you never get the credit.*

*It’s enough to drive you crazy if you let it”. – ‘9 to 5’ by Dolly Parton (1980)*

This excerpt from the famous Dolly Parton song *9 to 5* nicely captures one perception of the daily reality for many living in industrialized societies. Our modern way of living undoubtedly conveys many benefits, like the availability of commodities and an easy access to – say – job opportunities, healthcare or consumer goods. However, this lifestyle also has its dark side. Stressful living conditions have led to an increase of “modern” psychological and physical conditions, including cardiovascular diseases, obesity, diabetes or burnout [1]. Given these health impacts, many governments have understandably attempted to raise public awareness of, and institute various measures to prevent the deleterious health effects associated with life in modern industrialized societies. Also from a marketing perspective health (psychological and physical) has been picked up as a major selling point for the promotion of consumer goods [2].

Adopting a healthy diet and engaging in physical exercise are perhaps amongst the most straightforward preventive measures against the stress and strain of modern work and family life [3]. There is however also growing evidence that (passive) contact with natural environments can provide a psychologically “restorative” intervention. Specifically, restorative environments research has shown that exposure to nature, by – for example – taking a walk in the forest, or by looking out onto a patch of greenery from one’s window, can reduce stress [4], [5], negative moods [4], negative feelings (e.g., anger [6], [7]) and attentional fatigue [8], [9] and can also lead to increased (psychological) vitality [10]. These effects appear to be mostly limited to (unthreatening) natural environments, although a few studies have demonstrated superior restorative effects of (unthreatening) urban [11] and other artificial settings (e.g., museum [12], monastery [13]).

The theoretical backdrop for this paper on restorative nature experiences is Attention Restoration Theory (ART [14], [15], [16]). According to ART, the restorative effects of nature are situated in the fact that contact with unthreatening natural environments helps to recover attentional resources in individuals, especially when they are attentionally fatigued. One of the key reasons that natural environments are deemed to be generally more restorative than urban settings, is that they are a source of fascination [14]. As will be outlined in detail further on, three core dimensions are commonly attributed to the construct of fascination, as understood in the ART framework. Specifically, within ART fascination with nature is considered (a) to imply an *attentional bias* towards natural environments, (b) to be a relatively *effortless* mode of attention, and (c) to have *positive affective* valence by being an aesthetically pleasurable experience. Of these three dimensions the “effort” dimension (i.e. (b)) has received most consideration in the ART literature. The focus on effort is because restorative experiences are thought to hinge on the relative effortlessness with which natural environments are visually processed. Specifically, environments that support individuals functioning in a relatively effortless mode can provide – if needed – an opportunity to replenish depleted attentional resources.

Despite the fact that the three dimensions of fascination are thought to be the main drivers of restorative nature experiences, they have remained surprisingly underexplored within the ART framework. This is first of all clear from the fact that some of these dimensions have never been completely verified. Consider the claim that attending to fascinating natural environments is “effortless”. As far as we know, this assumption has barely been tested (but see [17]), but is only *derived* from the finding that there is a pre- to post-experimental improvement in performance on effortful attentional tasks, after exposure to natural as opposed to urban environments. Similarly, the contentions that unthreatening nature has positive affective valence, and that there is an attentional bias to those natural environments have only been scarcely tested within the ART framework (see [18] for an exception).

A second issue is that within the ART framework there is a tendency to measure the dimensions of fascination by means of self-reports, for example, with particular items of the Perceived Restorativeness Scale [19] or with adaptations of that scale [20]. Sample items from these scales are, for example, “*My attention is drawn to many interesting things*”, “*I want to get to know this place better*” or “*There is much to explore and discover here*”. However, as is well-known, explicit self-reports are prone to socially desirable answering, obscuring people’s actual and implicit attitudes towards natural environments. Another limitation is that the correlations between items that are supposed to be linked to restorative experiences could instead be due to employing one common method of measurement for all these items [21]. Thus even when self-reports can *prima facie* test whether individuals experience the three dimensions of fascination after exposure to nature, the fact that these measurement methods have not been complemented with more implicit and/or objective methods casts doubt on the validity and generalizability of these self-reports.

The general goal of this paper is to address the under-exploration of the dimensions of fascination in unthreatening natural environments. In order to investigate these dimensions we aimed to develop and employ measurement instruments that could more objectively capture these dimensions than introspection-based methods, such as self-reports. The instruments used are new to the field of restoration studies and are: (a) the affect misattribution task, (b) the dot probe paradigm and (c) a cognitive effortful task, which are aimed to respectively test the affective, attentional and effort dimensions. We anticipate that the use of these more objective instruments will methodologically enrich the field of restorative environments. Furthermore, their use is also expected to advance theory in this field, by verifying and further fine-tuning our understanding of some of the key-processes and characteristics assumed to underlie restorative nature experiences.

Note that we are aware that “objective” measurement instruments are sometimes used to test the restorative effects of contact with nature, as a complement to self-report measures, such as the Sustained Attention to Response Test (SART [9]) or the Attention Network Test (ANT [22]). Instruments like the SART and ANT are, however, primarily aimed at establishing whether, and the extent to which (depleted) attentional resources become restored after exposure to natural versus urban environments. In contrast, our use of more objective instruments is not motivated by the wish to test whether or not restoration has occurred. Rather, with our instruments, we have aimed to verify whether unthreatening natural environments are indeed characterized by the three dimensions that are ascribed to them within the framework of ART. For this verification, no prior depletion of attention is necessary, because if the three dimensions truly drive the process of restoration, they should then occur independently from depletion. A final difference is that in the studies reported here we will probe for the three restorative dimensions *during* environmental exposure. This differs from the common practice in restoration research of measuring the dependent variable of interest (i.e., attentional performance) *after* environmental exposure and will allow us to get a more direct insight into the processes that underpin restorative nature experiences.

This article is structured as follows. We first review the two main theoretical strands that make particular claims about the mechanisms through which nature is restorative. In the sections that follow we explain how exactly the construct of “fascination” is thought to contribute to the occurrence of restorative nature experiences. More specifically, we discuss the particular restorative dimensions that have been suggested to underlie fascination (i.e., the effort, attentional, and affective dimensions), review research that has attempted to measure these dimensions, and identify open questions. After this, the results of three studies are reported in which we attempted to more objectively test whether the suggested dimensions are indeed characteristic to visual encounters with (unthreatening) natural as opposed to urban environments. The paper concludes with a general discussion, in which the main results of these three studies, as well as their shortcomings, are discussed and related to the literature on restorative nature experiences.

### What Makes Natural Environments Restorative?

Up until this day, two theoretical frameworks have been central to restorative environments research, each of which highlights a particular aspect of restorative nature experiences. According to Stress Recovery Theory (SRT), natural environments are restorative primarily in their ability to trigger positive affect, which can dampen negative moods and provide a break from (negative) stress and arousal [23]. In recent years, however, ART has become the main theoretical basis that is used to explain and explore the beneficial effects of interacting with nature [14], [15], [16]. ART also forms the theoretical backbone of this paper. While SRT emphasizes the direct affective effects of contact with nature, ART adopts a more “cognitive” approach to restoration and states that nature is restorative by its ability to recover the capacity to direct attention. Directed attention is a capacity controlled by the central executive, recruited during tasks that require focus and concentration (e.g., proofreading), under voluntary control, demands effort, and requires the inhibition of competing activities and tasks [15], [16].

A crucial (but speculative) feature of directed attention is that it taps into – or is – a limited resource. Much like a muscle, this resource can run out of energy when it has been used too intensively for a prolonged period of time [16], leading to directed attention fatigue (DAF). The central tenet of ART is that natural, as opposed to urban environments, offer better opportunities to replenish this limited resource, and can therefore better alleviate DAF. It is claimed that natural environments have four “restorative” components that facilitate the process of restoration [14], [15]. Firstly, contact with nature can give individuals a sense of being away – either mentally or physically – from potential triggers of directed attention fatigue (“being away”). Secondly, natural settings are often rich in scope, distracting the mind from elements or events that can burden directed attention. At the same time, however, these environmental elements are still sufficiently connected to see the environment as an integrated whole (“extent”). Thirdly, in restorative environments there often is compatibility between an individual’s behavioral inclinations and intentions and the particular demands of the environment, making sure that less directed attention is needed to behave appropriately in the environment (“compatibility”). For example, a walk through a forested path demands little of the walker other than the task of walking itself.

The fourth component is fascination and the three studies reported in the current paper relate to three dimensions that are assumed to underlie the restorative power of fascination. Within the ART framework natural elements or scenes are considered to be intrinsically more fascinating, or to contain many more fascinating features or elements than urban environments. Fascination for nature is often visually based and can derive from many different visual sources, such as the rich and colorful textures of natural objects and environments (e.g., a field of flowers), the intricate fractal qualities of natural scenes, the presence of life (e.g., a flock of birds flying over), or the constant change that is often present in nature and natural elements (e.g., the flow of rivers or changing cloud patterns).

Although the construct of fascination forms a starting point for this paper, it is not our main focus. Rather, we will concentrate on the three constituent and interlocking dimensions of fascination, each of which contributes in some way towards the occurrence of restorative nature experiences. The first dimension is that fascination entails an attentional bias to the fascinating stimulus, or as Kaplan and Kaplan [14] frame it, fascinating things “attract people and keep them from getting bored” (p. 184). Therefore, by being a (stimulating) distraction, an unthreatening natural environment can turn one’s attention away from potential sources of DAF over an extended period of time.

The second dimension of fascination is that attending to natural objects or environments requires little cognitive effort [14], [15], [16]. This relative effortlessness assures that the resource, which is being expended when directed attention is recruited, is no longer overly stressed and can gradually recharge. As already noted, in ART natural environments are considered to be far more effortless to attend to than urban environments. The impact on effort of urban environments is thought to lie in the fact that these often contain many stimulating elements that compete for attention (e.g., traffic, people, billboards, and so on). Blocking out this urban (over)stimulation can actually hamper restoration, because the act of blocking these influences requires voluntary attentional effort, and thereby further stresses the capacity to directed attention [16].

One puzzle for ART is that some (fascinating) objects or processes can recruit attention in an effortless way, but they can hardly be considered as restorative – think for example of being threatened by a predator. Therefore, within ART, a further distinction is often made between two types of fascination, namely “hard” and “soft” fascination [14], [15], [16], [24]. Events or elements that trigger hard fascination (e.g., noticing a snake in the grass) grab one’s attention in an effortless way, but such experiences have a negative affective valence. Unthreatening natural environments (e.g., forests, parks), on the other hand, are reckoned to be softly fascinating, because they provide an affectively pleasant experience. Positive affect is the third dimension of fascination (besides the “effort” and “attentional” dimension), and it is assumed to complete the process of restoration by attenuating the unpleasantness that might arise from thinking about serious (life) issues [14].

### Measuring the Dimensions of Fascination

Being fascinated by natural environments both has “hot” and “cold” dimensions. The “cold” dimensions are the assumption that unthreatening natural environments attract attention, along with the claim that attending to such natural environments is relatively effortless. The “hot” dimension of (soft) fascination relates to the proposition that encountering unthreatening natural environments is generally an affectively or aesthetically more positive experience than attending to urban environments. In the following sections we review how each of these dimensions– i.e., “positive affect”, “effortlessness” and “attention” – has been measured both within and outside the field of restorative environments research.

### Affective Dimension

Although subjectively experienced positive affect toward natural settings seems to be an indicator of soft fascination, only little attention has been paid to positive affect within the context of ART. When positive affect is examined, it is mainly via self-reports, with natural environments leading to higher scores on positive affect than their urban counterparts [25]. The situation differs somewhat within the SRT framework. Here, positive affect has been researched both more frequently and in more objective ways. A priming experiment by Korpela and colleagues [26], for example, showed that respondents reacted faster to vocal expressions of joy than to expressions of anger after having viewed natural as opposed to urban settings. Similarly, happy faces were recognized faster when respondents had been primed with images of vegetated settings as opposed to images of built environments [27].These findings provide some indirect support for the claim that unthreatening natural scenes are experienced as affectively more positive than common urban scenes.

Also in research on human-nature interactions from outside the field of restoration studies, positive affect toward natural scenes and objects has been measured with more objective means than self-reports. For example it was found that after exposure to preferred natural scenes, activity in the zygomaticus major (facial) muscle increased, which was indicative of positive affect [28]. Also, a field study by Haviland-Jones and colleagues [29] showed that flowers were a source of positive affect because they triggered Duchenne smiles (i.e., “genuine” smiles) in the individuals to whom they were offered as a gift (compared to the individuals that were given another object, e.g., a pen as a gift). In our first study below, we will use another objective measurement to capture the positive affective dimension of softly fascinating natural environments. Specifically, we will introduce and employ the Affect Misattribution Procedure as a potentially useful instrument to track individuals’ implicit affective attitudes to natural versus urban environments.

### Attentional Dimension

Much like for the affective dimension self-report measures have mainly been used to explore the attentional dimension of fascination within ART (cfr., the perceived restoration scale (PRS) item: “*My attention is drawn to many interesting things*” [19]). Some restoration researchers have however attempted to get more direct and objective insight into individuals’ attentional functioning when perceiving unthreatening (natural) environments. Berto and colleagues [18], for example, explored whether individuals’ eye-movements differed for watching either urban or natural scenes. They found that the eyes of their participants covered significantly more space (i.e., produced more “saccades”) in the urban rather than the natural scenes, and the number of eye fixations was also higher in the urban condition. Note that this result prima facie speaks against the claim that fascination with nature entails an attentional bias to natural environments. Based on that view one would expect the natural scenes to cause the most saccades and fixations. Consider in this regard the PRS item for fascination, already mentioned before “*My attention is drawn to many interesting things”*. Would an environment with many things, which are interesting, not lead to more fixations and saccades? Note however that Berto and colleagues [18] interpret their results as evidence for the effortlessness of fascinating natural environments.

The claim for an attentional bias towards natural environments – as is made within ART – is consistent with some research findings from outside the field of restorative environments studies. Experimental psychology studies for example show that individuals preferentially attend to, or spend more time attending to particular categories of natural stimuli, and have an attentional bias to natural items as opposed to man-made objects. It is for example widely known that images of snakes presented among distractor images attract attention very rapidly [30]. Recent research, however, has shown that such an attentional bias also extends to nonthreatening life-like categories and processes. Specifically, Pratt and colleagues [31] found that biological motion captured visual attention more rapidly than nonbiological motion, which is suggestive of an attentional bias to particular characteristics of natural objects. In another example, New, Cosmides and Tooby [32] reported that respondents were faster and more accurate in detecting changes to scenes containing animals than to changes in scenes with inanimate objects, such as vehicles, which is again consistent with the claim for an attentional bias to natural stimuli. Eye-tracking studies also show that respondents are more likely to attend to animals than to man-made objects, and that animals are also attended to longer than to objects [33].

While these findings from outside the field of restoration studies seem to be consistent with the attentional dimension of ART, ART-based studies themselves have hardly investigated this assumption in a more objective fashion (except for the Berto et al. study [18]). Furthermore, these experimental psychological results have mainly been obtained with animal related stimuli as the “natural” stimuli. In contrast, in restorative environments research pictures of scenes dominated by vegetative elements, but devoid of animals, are typically employed as nature stimuli. This cast doubts on the generalizability of these experimental psychology results to the field of restoration studies. Based on these outstanding issues, Study 2 will introduce and employ the Dot Probe Paradigm as a more objective instrument to test the claim that people more readily attend to natural as opposed to urban scenes.

### Effort Dimension

The third restorative dimension of fascination is that fascinating (unthreatening) natural scenes are more effortless to attend to than (unthreatening) built, or artifact-dominated settings. Some self-report measures tap into this specific restorative dimension, such as the item “*There are many things here that attract my attention effortlessly*” (item taken from the Perceived Restorative Characteristics Questionnaire (PRCQ) [20]). However, to our knowledge, only one attempt within restoration research has been made to test the effort dimension of fascination in a direct, objective manner. This attempt was made by Berto and colleagues [17] who found that attentionally fatigued respondents were more quickly able to make attentional shifts when they had natural as opposed to urban environments in their visual field. The authors explained this result in terms of the supposed effortless mode of attention that is supported by natural environments. Most often, however, the “effort” dimension of fascination is only *inferred* from experimental results. For example, it is derived from the fact that attentionally fatigued respondents score better on tasks that require directed attention when, before the task they have been exposed to natural as opposed to urban environments [34].

In some cases findings from psychological studies from outside the field of restoration studies have also provided (circumstantial) support for the claim that natural versus artifactual stimuli are more effortless to process. For example, individuals have been found to categorize natural scenes faster than scenes dominated by man-made elements, which is *prima facie* consistent with the claim that natural scenes are more effortless to process than man-made environments [35], [36]. Some vision research experiments have further demonstrated that the functioning of the human early visual brain closely matches the specific statistical (i.e., fractal) properties of natural scenes, which has been taken by some as (indirect) evidence that the early visual system is optimized for sparsely coding, and effortlessly processing the visual properties of natural environments [37].

However, one of the issues with the previous research is that the visual stimuli that were used often do not fall along the same lines as those used in restoration research. For example, in the Rousselet, Joubert and Fabre-Thorpe [35] study, pictures of natural scenes also included spectacular nature, such as dramatic mountainscapes. In restoration research spectacular nature is however thought to have relatively little restorative potential because it is a source of hard rather than soft fascination. In sum, there is need of experiments that squarely fall within the ART framework and that try to more objectively assess the supposed effortlessness of attending to natural versus urban environments. With Study 3 we aimed to address this issue by using an effortful recognition task, during which participants were exposed to pictures of either urban or natural environments.

### Overview of the Studies

Empirical research, situated within the framework of ART, often relies on subjective and derivative measures to investigate the three dimensions of soft fascination with natural environments (i.e., positive affect, attention, effortlessness). Using self-reports has obvious advantages, such as the fact that substantial amounts of information can be obtained from large samples of respondents in a relatively short period of time. However, research shows that especially with regard to people’s relationship with the natural world, there often is a gap between their expressed attitudes towards it (e.g., willingness to recycle) and their actual behavior (e.g., actual recycling behavior) [38]. In a similar way, it can be questioned whether people’s self-reported fascination with nature converges with actual, more implicit indices of fascination. Importantly, although it has been hypothesized that the effort, attentional and affective dimensions of fascination (at least partly) underlie restorative nature experiences, surprisingly little research has been dedicated to settling whether or not these dimensions actually apply to individuals’ experiences of natural environments. Is it truly the case that unthreatening nature more readily attracts attention, is more affectively pleasing, and more effortless than common urban environments? The central aim of the ensuing studies is to take a step back, and to try to empirically address these questions with more objective measurement instruments than self-reports. With these new tools, and the insights we derive from their use, we hope to advance our understanding of the mechanism(s) through which the process of restoration unfolds.

### Study 1

The first study aimed to test the hypothesis that unthreatening natural environments are experienced as affectively more pleasant than their unthreatening urban counterparts by means of the affect misattribution procedure (AMP). The AMP can provide insight into individuals’ implicit affective attitudes towards certain stimuli, by tapping into the tendency to misattribute the feelings triggered by those stimuli to affectively neutral stimuli.

### Study 2

The second study employed the dot probe paradigm (DPP) to test the hypothesis that unthreatening natural environments more readily attract attention than nonthreatening urban environments. The DPP provides insight into where individuals allocate their visual attention by showing them two (attentionally competing) images and by monitoring their performance on a subsequent spatial locating task. (Note that Study 2 and Study 1 were part of one large overall study, and were performed with the same participants. Both studies will, however, be discussed separately in the ensuing sections).

### Study 3

With the third study we aimed to test the hypothesis that natural scenes are less effortful to attend to than urban scenes. For this purpose, respondents performed a cognitively effortful task while they *simultaneously* had a picture of either an urban or a natural environment in view. If natural scenes are indeed less effortful to attend to than urban scenes, we should find that respondents perform better – both in terms of speed and accuracy – on the task when they have natural as opposed to urban environments in view.

### Environmental Picture Set

**Figure 1:**
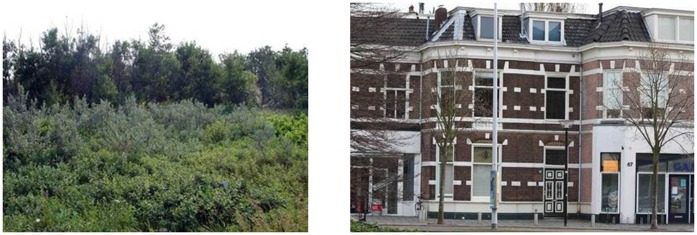
Two sample pictures of the stimulus set.

The set of environmental pictures that we used across all three studies consisted of fifteen pictures of urban settings and fifteen pictures of natural settings (see Figure 1 for sample pictures). This picture set included digital photographs collected from the internet, as well as photographs taken by one of the authors. The natural images depicted (unthreatening) vegetated environments of varying openness, whereas the urban pictures mainly showed (unthreatening) streetscapes with buildings of different architectural styles (e.g., family dwellings, traditional and modern buildings). Care was taken that there was sufficient variation in the aesthetic qualities of the scenes of each image set. Specifically, in both image sets the selected pictures ranged from being very mundane (e.g., grey building façade; thicket) to depicting relatively pretty natural/urban settings (e.g., a forest in spring; a building with decorated façade). In Study 1 and 2 this entire picture set was used as stimulus material, whereas in Study 3 three pictures of each condition were left out, to avoid excessive cognitive fatigue in respondents.

## Study 1

The aim of the first study was to test the affective dimension of fascination, i.e., the hypothesis that people find unthreatening natural environments affectively more pleasant than unthreatening urban environments. This hypothesis was tested using the AMP, which is able to capture individuals’ implicit affective evaluations of stimuli [39], [40], [41]. The AMP provides a more objective alternative to the self-report measures that are typically employed in restorative environments research and, at the same time, it reduces the likelihood of common method variance.

In the AMP, a picture is first shortly, but visibly shown to participants, directly followed by what is considered to be the “target” stimulus, which most often is an affectively neutral Chinese pictograph. Participants are instructed to neglect the first picture, but to evaluate the visual pleasantness of the Chinese pictograph. As participants have difficulty in disentangling their affective responses to two events occurring in close proximity in time and space they are inclined to misattribute some of their implicit affective evaluations of the first picture to the Chinese pictograph.

In our version of the AMP the pictures that were presented before the Chinese pictographs were pictures of either natural or urban environments. Based on the hypothesis that (unthreatening) natural environments are more affectively pleasant than (unthreatening) urban environments [23], participants were expected to evaluate the Chinese pictographs that followed a natural picture as more pleasant than those that followed an urban picture.

### Methods

#### Ethics statement

Ethical approval for running the experiment was obtained from the ethical commission of the Department of Psychology, University of Groningen (contact: ecp@rug.nl; committee: Peter de Jong (committee chair), Christine Falter, Yvonne Groen, Eric Rietzschel). Participants provided their written informed consent to participate in this study. This consent procedure was approved by the ethics committee.

#### Participants and design

Ninety-five first-year psychology students from the University of Groningen (29 males) participated in this study in exchange for course credit. Due to a programming error, data on the age of the respondents were lost in the three studies. We estimate that, given the fact that it was a student sample, the age ranged from 18 to 25 years old. In the experiment we manipulated the environmental stimuli, i.e., images of natural versus urban environments, as a within-subjects variable.

#### Material and procedure

The material in this computer-based experiment (programmed using E-prime) consisted of (i) target stimuli and (ii) environmental pictures. The target stimuli were thirty different Chinese pictographs that were collected from the internet. The environmental pictures (N = 30) were fifteen urban and fifteen natural images, belonging to the picture set that was used across all three studies.

On arrival in the lab each participant was guided to a personal computer and filled out an informed consent form. Participants were verbally informed that they would see pictures followed by Chinese pictographs. Before the experiment started, the specific instructions for the AMP were presented to the participants on the computer screen. They were told that the presentation of the environmental pictures was merely to prepare them for the upcoming Chinese pictograph. Their task was to focus on the pictographs and to evaluate the visual pleasantness of each of them. Specifically, participants were instructed to press the “z” key on their keyboard when they found the Chinese pictograph “*less pleasing than average*” and the “m” key when they found the pictograph “*more pleasing than average*”.

**Figure 2:**
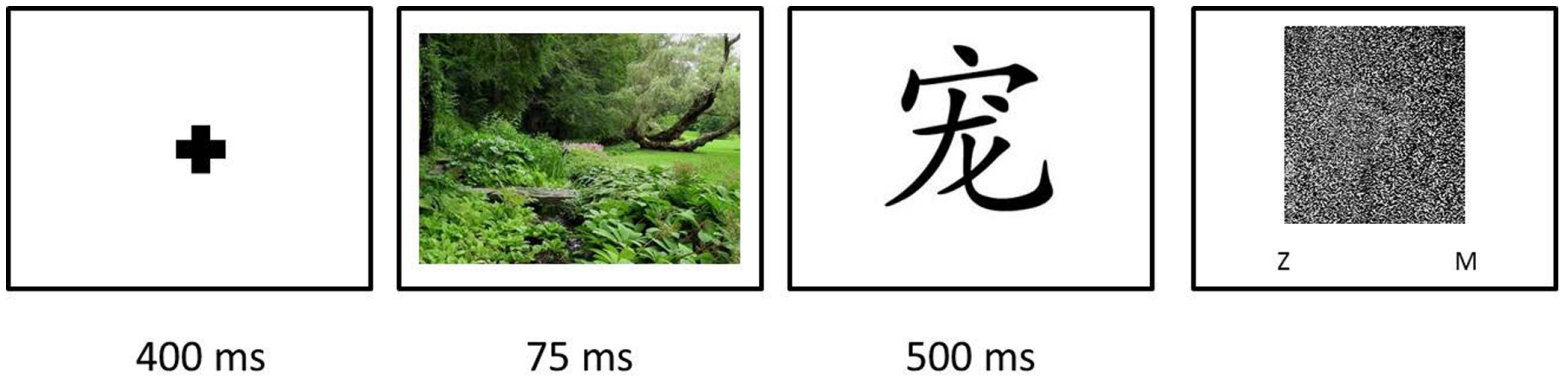
Flow of the affect misattribution task.

Before the actual experiment started, participants completed ten practice trials that were identical to the experimental trials, except that the images preceding the Chinese pictographs were of neutral objects (e.g., cars, bicycles and furniture). After completing these practice trials, a screen appeared to inform participants about the upcoming experimental trials. Each trial of the AMP started with a fixation point that appeared on the center of a white screen for 400 milliseconds. After that, an environmental image – either “urban” or “natural” – was displayed for 75 milliseconds. Each image was randomly selected from the set of thirty environmental stimuli. Following this, a white screen appeared for 125 milliseconds, after which a Chinese pictograph was presented for 500 milliseconds. The final screen consisted of a noise square in the location of the previously shown pictograph, and the two possible responses to the visual pleasantness of the pictograph (“z = less pleasing than average” and “m = more pleasing than average”). The next trial started as soon as the participants had evaluated the pictograph (Figure 2). Participants completed thirty randomly ordered trials, including fifteen urban pictures and fifteen natural pictures as pre-targets, and thirty different Chinese pictographs as targets, each of which were only presented once and were selected at random for each trial. The pairing of each unique pictographs with each unique urban or natural image was thus also random.

A number of calculations were performed on the dataset, yielding two categories of dependent variables. First, for each participant we determined the total number of affective evaluations for each environmental picture class. This resulted in four values:

- **POS_nature_**: Number of positive evaluations of the pictographs preceded by natural images.
- **NEG_nature_**: Number of negative evaluations of the pictographs preceded by natural images.
- **POS_urban_**: Number of positive evaluations of the pictographs preceded by urban images.
- **NEG_urban_**: Number of negative evaluations of the pictographs preceded by urban images.

The second category of dependent variables was the overall affective score for each picture class. This variable was calculated by subtracting the total score of each individual’s negative evaluations (e.g., “NEG_urban_” for the urban picture class) from his/her total score of positive evaluations (e.g., “POS_urban_” for the urban picture class) for that class. This resulted in a score ranging from −15 to +15, capturing an individual’s overall mean affective evaluation of the pictographs associated with one picture class (e.g., urban). A score of “−15”, for example, indicated that all affective evaluations of the pictographs preceding the images of that picture class were negative, while a score of “+15” meant that all responses to the pictographs were positive.

### Results and Discussion

Analyses of the data-set were performed with SPSS statistical software. Using paired samples t-tests we first looked at whether, for one picture class (e.g., “nature”), participants differed in the average number of positive (e.g., POS_nature_) versus negative affective evaluations (e.g., NEG_nature_) of Chinese pictographs. Our analyses showed that when Chinese pictographs were preceded by nature pictures, on average, the scores for POS_nature_ (*M* = 10.42, *SE* = 0.43) were significantly higher than the scores for NEG_nature_ (*M* = 4.58, *SE* = 0.43), *t*(94) = 6.72, *p*<.001, *r* = .56). A similar result was obtained when the Chinese pictographs followed urban images. Here, participants on average scored significantly higher on POS_urban_ (*M* = 8.51, *SE* = 0.34) than on NEG_urban_ (*M* = 6.49, *SE* = 0.34), *t*(94) = 2.90, *p* = .005, *r* = .28. The Chinese pictographs thus received more positive affective evaluations than negative ones overall, no matter if they were preceded by images of either urban or natural settings.

To test the hypothesis that natural scenes feel affectively more pleasant than urban scenes, we looked at possible differences between respondents’ overall affective scores for either the “urban” or the “natural” picture class. A paired samples t-test revealed that the mean affective evaluations were more positive when the Chinese pictographs were preceded by nature images (*M = *5.84, *SE = *0.86) than when they were preceded by urban images (*M = *2.01, *SE = *0.69), *t*(94) = 5.14, *p*<.001, *r* = .46. This finding is consistent with the affective dimension of fascination, which suggests that unthreatening natural environments are experienced as affectively and aesthetically more pleasant than unthreatening urban environments and therefore are more likely to be (softly) fascinating.

## Study 2

The aim of the second study was to test the attentional dimension of fascination, i.e., the hypothesis that (unthreatening) natural environments more readily attract visual attention than (unthreatening) urban environments. We tested this hypothesis with an adapted version of the Dot Probe Paradigm [42]. The DPP was originally developed to demonstrate individuals’ selective attention to threatening as opposed to neutral stimuli. The DPP starts off with simultaneously, and very shortly, presenting two stimuli side by side on a computer screen. After that, a probe – often a small dot – appears on one of the two screen locations of the preceding two stimuli. Participants are instructed to try to detect the dot as quickly as possible by pressing a pre-specified button that corresponds to the location of the dot on the computer screen. If visual attention has been attracted or captured by one particular stimulus of the previously presented pair this will result in quicker detection of dots appearing in the same region of the computer screen as the attended stimulus, and slower detection of dots appearing in a different region [43].

Since its development, the DPP has been used to demonstrate individuals’ attentional bias to other types of stimuli than to threats. For example, it has been employed to show that smokers have an attentional bias towards smoking-related images [44], and that hungry people are attentionally biased towards food-related words [45]. In the current study, we used image pairs of urban and natural scenes. In agreement with the claim that unthreatening natural environments more readily grab attention than their unthreatening urban counterparts, we hypothesized that in the DPP, the dots would be detected more rapidly when they were located in the former location of the natural, as opposed to the urban pictures.

### Methods

#### Ethics statement

Ethical approval for running the experiment was obtained from the ethical commission of the Department of Psychology, University of Groningen (contact: ecp@rug.nl; committee: Peter de Jong (committee chair), Christine Falter, Yvonne Groen, Eric Rietzschel). Participants provided their written informed consent to participate in this study. This consent procedure was approved by the ethics committee.

#### Participants, design and material

Eighty-nine first-year psychology students from the University of Groningen (29 males) participated in this study in exchange for course credits. In this study, DPP data from 6 participants were not saved due to an error, which explains why the number of participants in this study differs from the number of participants in Study 1. The same environmental stimuli as in Study 1 were used (i.e., fifteen images of natural and fifteen images of urban environments) and these were manipulated as a within-subjects variable.

#### Procedure

On arrival in the lab each participant was guided to a personal computer, filled out an informed consent form, and was verbally informed about the upcoming experiment by the experimenter. Before the actual DPP started, participants completed ten practice trials of the DPP, which were identical to the experimental trials except that the image pairs were of neutral objects (e.g., cars, bicycles and furniture).

**Figure 3:**
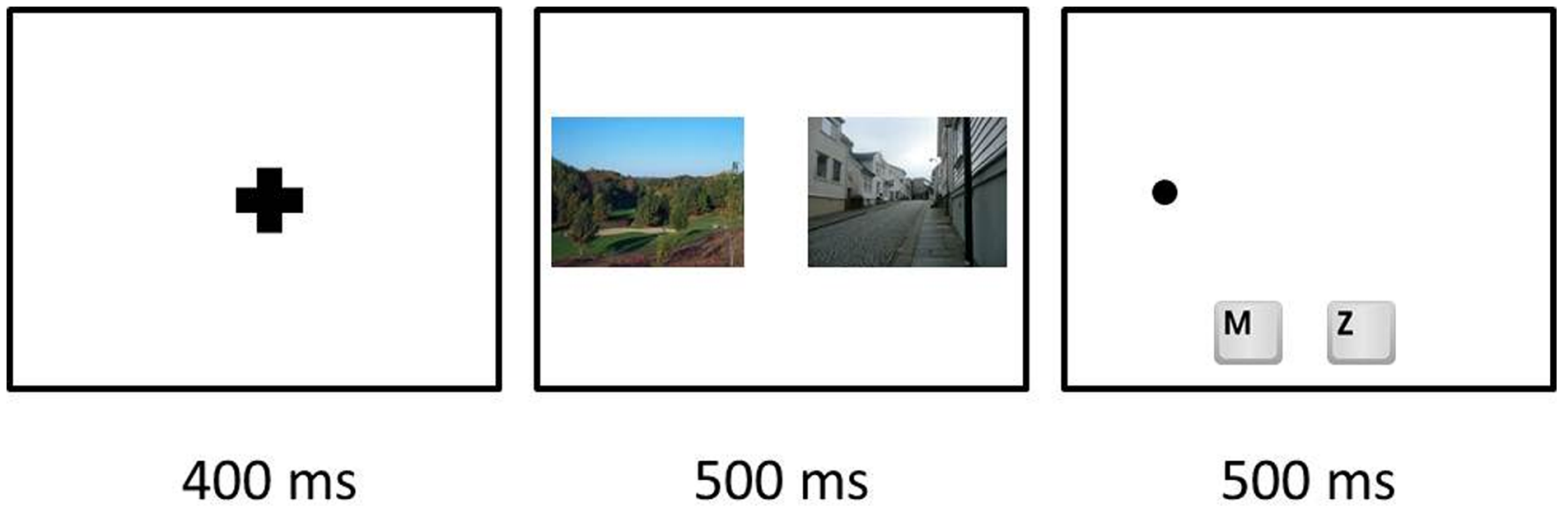
Flow of the dot probe task.

At the beginning of the DPP a fixation point appeared on the center of a white screen for 400 milliseconds. After this, an image pair was displayed on the screen for 500 milliseconds. One image was positioned on the middle of the right side of the screen, whereas the other one was positioned on the middle of the left side On each trial, the image pair consisted of one natural and one urban picture that were randomly selected from the original picture set. The position (left or right) of the two types of environmental pictures was counterbalanced across trials. In a number of the trials – i.e., the “valid trials” – a small dot appeared on one of the two screen locations that were previously occupied by one of the two environmental pictures (dots did not appear in all trials to prevent habituation and to keep the participants attentive). That dot-probe screen lasted for 500 milliseconds and participants had to react as fast as possible and press the “z” key when the dot appeared on the left side of the screen and the “m” key when it appeared on the right side (Figure 3).

In total, the participants had to perform a session of twenty experimental DPP trials. The number of valid trials that each participant could receive in a session (i.e., the trials where a dot appeared) was, however, not fixed. Rather, each participant was randomly assigned *x* number of valid trials, where *x* could range from 0 to 6. There were two types of valid trials, one where a dot appeared on the former location of the natural image (i.e., “valid nature trials”), and one where it appeared on the former location of the urban image (i.e., “valid urban trials”). (The reason why the number of trials was not fixed was because the DPP trials with environmental pictures were part of a larger DPP session that also included other types of pictures (e.g., fractal-like shapes). This entire session had a fixed number of valid trials, whereas *within* this large session the possible number of valid trials associated with the environmental pictures varied).

The study yielded two categories of dependent variables, for both the valid nature trials and the valid urban trials: (i) the time needed to respond to the location of the dot (in milliseconds) and (ii) the accuracy with which the dots were located. Only the response times for correct trials were considered and these corresponded to the time that elapsed from the moment a dot appeared on the screen until respondents had pressed the appropriate key. The accuracy with which participants located the position of the dots was calculated for both valid urban and valid nature trials. This was done by dividing the total number of correct responses to the valid trials by the total number of valid trials that were presented to each participant, with a value of “1” signifying complete accuracy.

### Results and Discussion

Analyses of the data-set were performed with SPSS statistical software. Because a limited number of participants had not received any valid trials at all (see the previous section), these analyses could only be performed for the participants who had received at least one valid nature trial and one valid urban trial (N = 78).Two participants were excluded from the analyses because they performed poorly in locating the position of the dots. They were excluded on the basis of outlying values for the accuracy of valid nature trials. Outlying values were defined as those that were equal to 0.

Participants were on average significantly more accurate in identifying the location of the dot in the valid nature trials (*M* = 0.83, *SE* = 0.02) than in the valid urban trials (*M* = 0.75, *SE* = 0.03), *t*(82) = −2.04, *p*<.05, *r* = .21. In addition, respondents reacted significantly faster (*M* = 412.82, *SE* = 5.08) when a dot appeared in the location where formerly a nature image had been positioned, than when the dot appeared in the former location of an urban image (*M* = 428.51, *SE* = 4.07); *t*(77) = 3.43, *p* = .001, *r* = .36. These results support our hypothesis that people are attentionally biased towards unthreatening natural as opposed to unthreatening urban scenes.

Note that including the two outlying individuals in the analyses only impacted overall accuracy (not response times), and made the difference between the accuracy for the valid nature trials (*M* = 0.81, *SE* = 0.02) and valid urban trials (*M* = 0.74, *SE* = 0.03) marginally significant; *t*(84) = 1.75, *p = *.08).

## Study 3

The goal of the third study was to test the effort dimension of fascination, i.e., the hypothesis that (unthreatening) natural environments are more effortless to attend to than (unthreatening)urban settings [14], [15], [16]. This hypothesis was tested by requiring that respondents carry out a series of cognitively effortful visual recognition tasks, while they were simultaneously exposed to pictures of either natural or urban environments. This task was developed to provide a more objective and reliable measure of cognitive performance than self-reported effort [20], as well as to measure (aspects of) participants’ cognitive functioning during, rather than after viewing environmental images. Two general predictions regarding task performance were derived from ART. First, if it is indeed the case that less cognitive effort is required to attend to natural environments than to urban environments, participants should be more accurate in solving the cognitively effortful task when natural as opposed to urban pictures occupy their visual field. Second, because increased cognitive effort negatively affects the speed of (cognitive) processing [46], the cognitively effortful task is expected to be more rapidly executed when respondents attend to the (supposedly “effortless”) pictures of natural as opposed to urban environments.

### Methods

#### Ethics statement

Ethical approval for running the experiment was obtained from the ethical commission of the Department of Psychology, University of Groningen (contact: ecp@rug.nl; committee: Peter de Jong (committee chair), Christine Falter, Yvonne Groen, Eric Rietzschel). Participants provided their written informed consent to participate in this study. This consent procedure was approved by the ethics committee.

#### Participants and design

Thirty nine psychology students from the University of Groningen participated in this study in exchange for course credits. In the experiment we manipulated the environmental stimuli, i.e., images of natural versus urban environments, as a within subjects variable.

#### Materials

The following materials were used in this study: (i) twelve photographs of natural environments, (ii) twelve photographs of urban environments, and (iii) twenty four sequences of geometrical shapes. The two sets of photographs were taken from the set of thirty photographs that was used in Studies 1 and 2. Slightly fewer pictures were used in the current study (twelve in each condition, instead of fifteen) to prevent respondents from becoming too cognitively fatigued.

**Figure 4:**
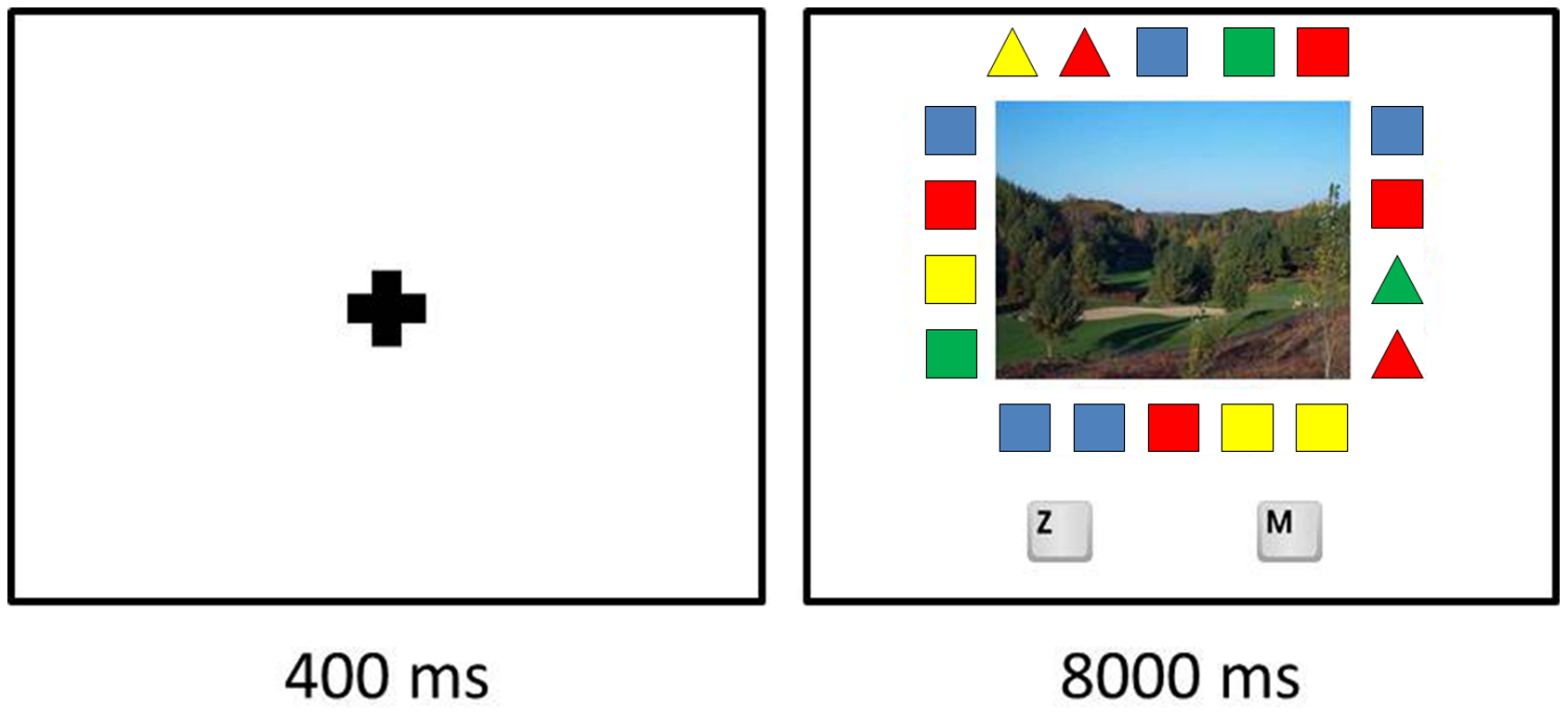
Flow of the cognitive effort task.

The environmental photographs were combined with sequences of simple geometrical shapes, which were created with *Microsoft Paint*. All sequences consisted of eighteen shapes – squares and triangles of different colors – which were arranged along the four sides of each environmental photograph (see Figure 4). Different types of sequences were created depending on whether or not they met one of the four following properties.

- *Property (a)*: the sequence contained exactly four triangles.
- *Property (b)*: the sequence contained exactly four triangles, and two of which were adjacent.
- *Property (c)*: the sequence contained exactly four triangles, two of which were adjacent, and each of the four triangles had a different color.
- *Property (d)*: the sequence did not have any of the above three properties.

Based on these four properties, two types of sequences were created, namely “target” and “nontarget” sequences. A “target” sequence was defined as meeting property(c) (and per definition thus also properties (a) and (b)). Three types of “nontarget” sequences were created, depending on whether they met one of the conditions outlined below.

- “Type I” nontargets: only meet property (d)
- “Type II” nontargets: only meet property (a)
- “Type III” nontargets: only meet property (b)

A total of twenty four sequences of geometrical shapes were created. Twelve sequences were arranged around the twelve nature images, and twelve were arranged around the twelve urban images, given a total of twenty four experimental stimuli. Each of the two sets of twelve sequences always contained six “targets”, and six “nontargets”. The six nontargets, in turn, consisted each time of two type I, two type II, and two type III nontargets.

We anticipated that the identification of a given sequence would require more cognitive effort when identification depended on checking for more properties. Based on this, we expected that overall recognition times would be slower for targets than for nontargets, and also within nontargets we expected that response times would become slower as more properties needed to be checked. For example, a nontarget sequence without any triangles at all is easier to detect as being a nontarget than a nontarget consisting of four triangles, two of which are adjacent, and two having the same color. Including varying levels of difficulty (i.e., identifying more or less properties) allowed us to explore whether there is a particular threshold (of cognitive effort) at which the hypothesized “nature effect” becomes outspoken.

#### Procedure

On arrival in the lab, each participant was guided to a personal computer, filled out an informed consent form, and was briefed about the upcoming experiment. During the experiment, participants were shown a series of experimental stimuli, and their task was to identify as fast as possible whether or not the sequence of shapes surrounding the environmental picture was either a target or a nontarget.

Before the actual experimental trials began, participants completed ten practice trials, with images of neutral objects (e.g., bicycles and cars) rather than of the urban or natural environments. Participants had to complete a total of twenty four actual experimental trials, and these were presented in two consecutive phases. In one phase they were shown the twelve experimental stimuli containing natural images, and in the other phase they viewed the twelve stimuli with urban images. The order of presentation of these two phases was counterbalanced among participants to control for order effects. Within each phase, targets and nontargets were randomly presented to the participants.

The experimental trials always started off with a fixation point that remained in the middle of the screen for 400 milliseconds, preparing the participant for the upcoming stimulus. After that, an environmental picture, surrounded by a sequence of geometrical shapes, appeared. Participants were instructed to indicate whether the sequence was a target (by pressing the “z” button on the keyboard) or a nontarget (by pressing the “m” button). They had to make this decision as fast as possible, the time limit for their response being 8000 milliseconds. When all twelve trials associated with one category of environmental pictures was completed, a white screen appeared for 1000 milliseconds. After this, the twelve trials associated with the other category of environmental pictures were presented.

The study yielded two categories of dependent variables for both the natural and urban experimental stimuli: (i) the overall accuracy with which participants identified targets and nontargets, and (ii) the time needed to correctly identify the sequence as either a target or a nontarget (in milliseconds). Accuracy was the accuracy score for targets and nontargets summed together for either the natural or the urban condition. As everyone received twelve experimental stimuli (i.e., an environmental picture surrounded by a shape sequence) for each environmental condition, accuracy was determined as the number of correct identifications of targets and nontargets (with “0” being entirely inaccurate and “12” being fully accurate). Only the response times for correct identifications were considered and these corresponded to the time that elapsed from the moment the experimental stimuli appeared on the screen until respondents pressed the appropriate key.

### Results and Discussion

Analyses of the data-set were made with SPSS statistical software. Five participants were excluded from the analyses because of their poor performance in correctly identifying targets and nontargets. Participants were excluded on the basis of outlying values for the accuracy with which they identified sequences that were positioned around natural images. Outlying values were defined as those that were equal to, or smaller than 9.

We first looked at the time it took participants to identify targets and nontargets, irrespective of the image type around which the sequences were arranged (i.e., natural or urban) (see Table 1 for mean identification times). Paired samples t-tests revealed that targets were on average recognized significantly slower than type I (*t*(34) = 7.25, *p*<.001, *r* = .77), type II (*t*(34) = 8.44, *p*<.001, *r* = .82) and type III (*t*(34) = 6.90, *p*<.001, *r* = .76) nontargets. This is consistent with our expectation that less cognitive effort is required to identify nontargets than targets.

**Table 1:** Mean identification times (milliseconds) and standard errors for identifying Type I, Type II, Type III nontargets and targets.

	Type I nontarget		Type II nontarget		Type III nontarget		Targets	
	Means	standard error	means	standard error	means	standard error	Means	standard error
nature	2801.62	128.11	2803.41	130.05	2924.18	198.84	3112.00	130.39
urban	2088.10	123.00	2254.38	137.28	2311.78	153.12	3525.38	169.25
overall	2444.86	106.52	2528.90	115.71	2617.98	148.41	3318.69	1138.84

Considering the three types of nontargets, analyses showed that on average type I nontargets were recognized faster than type II nontargets, and type II faster than type III. However, statistical analyses showed that none of these differences was significant. Specifically, there were no significant differences in recognition time between type I and type II (*t*(34) = −0.92, *p* = .36), between type II and type III (*t*(34) = −0.80, *p* = .42), and between type I and type III nontargets (*t*(34) = −1.34, *p* = .18).

We then analyzed with paired samples t-tests whether there were any differences in recognition times of the sequences, depending on whether they were arranged around either natural or urban images. We first checked whether there were any differences in overall response time, i.e., the response times for all the sequence types (i.e., targets and nontargets) summed together. Analyses showed that, contrary to our expectations, sequences were identified significantly faster when surrounding an urban picture (*M* = 2544.91, *SE* = 113.80) than when surrounding a nature picture (*M* = 2910.30, *SE* = 120.71); *t*(34) = 4.47, *p*<.001, *r* = .60, suggesting that the urban rather than the natural scenes were more effortless to attend to.

**Figure 5:**
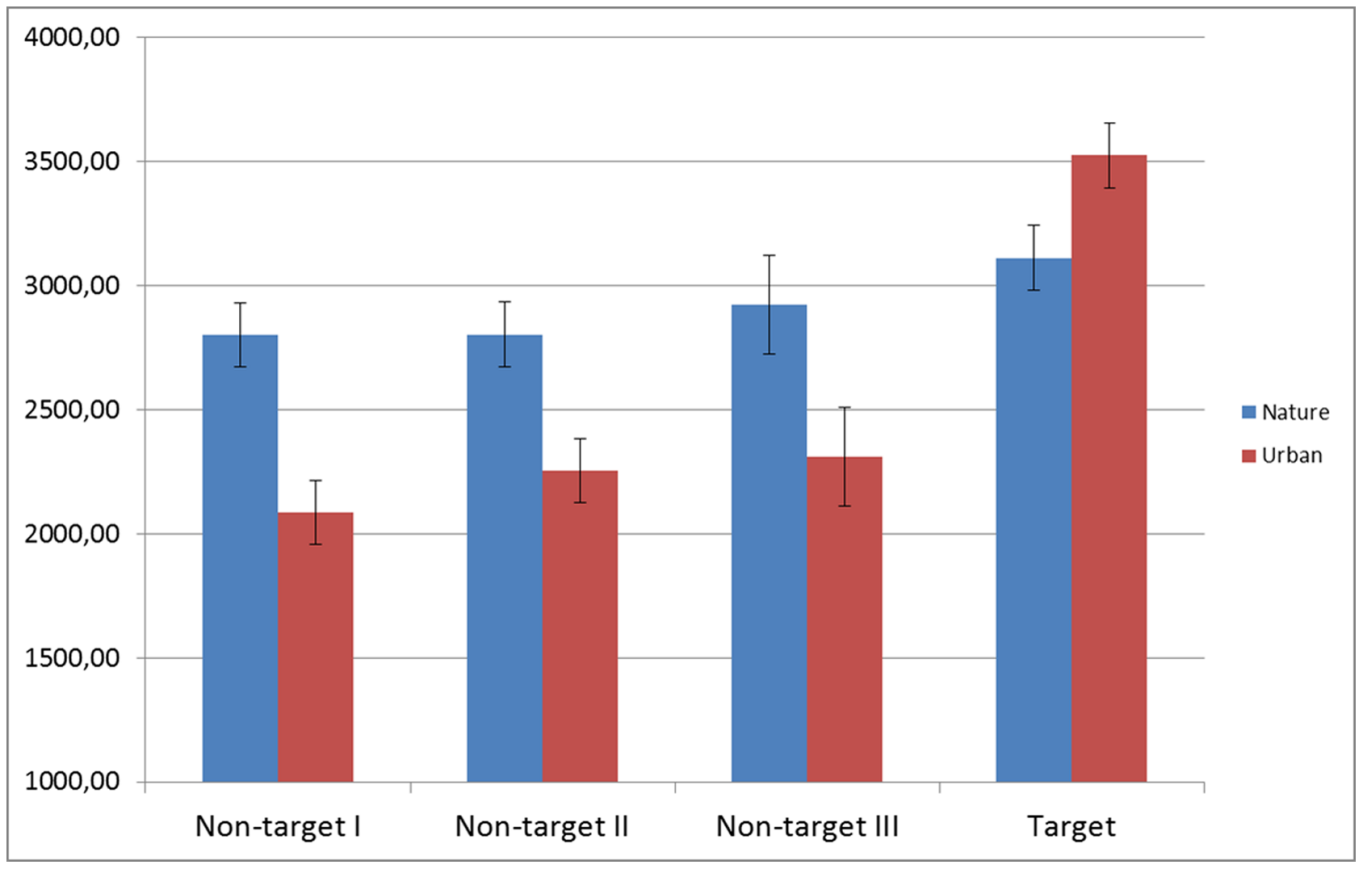
Graph of the identification times for targets and all types of nontargets across the urban and natural condition.

However, a more subtle picture emerged when we looked at response time differences for the different types of sequences (see Table 1 for identification times). For targets, we found significantly quicker recognition times when targets were arranged around the natural as opposed to the urban images; *t*(34) = −3.47, *p* = .001, *r* = .51. On the other hand, all three types of nontargets were more quickly identified when they were positioned around urban as opposed to natural images. These differences were statistically significant for type I (*t*(34) = 5.36, *p*<.001, *r* = .67), type II (*t*(34) = 4.09, *p*<.001, *r* = .57) as well as type III (*t*(34) = 3.14, *p*<.005, *r* = .47) nontargets. These results are visualized in Figure 5. What is noticeably is that for the “natural” experimental stimuli, the recognition time for all four types of sequences gradually increased as more properties needed to be identified. For the “urban” experimental stimuli, recognition times across all nontarget types were similar and fairly quick, but there was a steep “jump” in the recognition times of targets.

Finally, we looked at differences in the accuracy of identifying sequences, depending on the type of environmental image which they surrounded. A paired samples t-test reveals statistically significant differences (*t*(34) = 3.43, *p*<.005, *r* = .50) with higher accuracy when sequences were surrounded by natural (*M = *11.51, *SE = *0.09) as opposed to urban images (*M* = 10.91, *SE = *0.19). Participants were thus more accurate in identifying sequences when these were arranged around pictures of natural as opposed to urban scenes.

Note finally that including the five outlying participants in the analyses only impacted overall accuracy (not identification times), and made the difference between the overall accuracy for the sequences surrounding natural pictures (*M* = 11.13, *SE* = 0.18) and the accuracy for the sequences surrounding urban pictures (*M* = 10.93, *SE* = 0.18) nonsignificant; *t*(39) = 0.82, *p = *.41.

## General Discussion

According to ART unthreatening natural environments are restorative in large part because they are fascinating [14], [15], [16]. Being fascinated by natural environments promotes restorative nature experiences because fascination is assumed to be characterized by three core dimensions. Fascination (a) is a relatively effortless mode of attention, enabling attentional resources to replenish; it (b) implies an attentional bias to the environment, distracting a person’s attention away from potential sources of directed attention fatigue; and it (c) is a positive affective experience, creating relaxation and attenuating negative moods that might arise from thinking about important (life) issues.

As was pointed out throughout this paper, our understanding of these three dimensions has so far mainly relied on self-reports. However, nowadays concepts and/or messages referring to, or related to the natural world (e.g., “biological”, “organic”) are typically bound to lead to positive semantic associations in individuals, such as “purity”, “goodness” or “niceness” [47]. So although self-reports measurements might show that people experience nature as affectively pleasant (cfr., affect dimension) or that nature grabs our attention in an effortless manner (cfr., attentional and effort dimension), that may just be a reflection of widespread cultural beliefs, instead of accurately reflecting individuals’ actual experiences of nature. To address the potential shortcomings of self-reports we ran three experiments, which were aimed at more objectively verifying whether the experience of unthreatening natural as opposed to unthreatening urban environments is indeed effortless, affectively positive, and implies an attentional bias.

In Study 1 we aimed to more objectively test the affective dimension of fascination with natural environments by means of the affect misattribution procedure (AMP). In our version of the AMP, respondents were asked to rate the visual pleasantness of Chinese pictographs that were preceded by pictures of either urban or natural environments. We detected misattributions of affective attitudes towards the environmental pictures onto the pictographs. Specifically, as expected, the pictographs preceded by natural pictures received significantly more positive evaluations than those that were preceded by urban pictures. This suggests that on average the natural images were experienced as affectively more positive than the urban ones, providing objective evidence for the claim that unthreatening natural environments are experienced as affectively more positive than unthreatening urban environments [14].

The second dimension of fascination is that people are supposed to be more biased towards attending to unthreatening natural environments than to unthreatening urban environments. In Study 2 we tested this hypothesis by means of the dot probe paradigm (DPP). In our version of the DPP respondents were first shortly presented a pair of images, consisting of an urban and a natural image, for a short time period. After this the images disappeared and a dot appeared on the screen, and respondents had to locate the position (left or right) of that dot as fast as possible. In the DPP dots are identified quickly when they appear in the position that used to be occupied by a picture of an image pair that previously attracted the most attention. Our results showed that in the DPP, the dots were located the quickest when they were positioned on the former location of natural as opposed to urban images. This suggests that, on average, respondents were indeed attentionally biased towards the natural rather than to the urban images, which is in accordance with the attentional dimension of ART. We also found that respondents were most accurate overall in locating the dots when they appeared on the former position of natural as opposed to urban pictures. Perhaps this is due to the fact that locating a target is easier, and thus more accurate, when one’s attention is already directed to the position of the target than when it is not.

The third suggested dimension of fascination is that natural environments are more effortless to attend to than their urban counterparts. With Study 3 we attempted to test this dimension by tracking participants’ cognitive performance on a series of effortful tasks (i.e., recognizing target versus nontarget sequences of geometric shapes). In contrast to most research on restorative environments, the dependent variable of interest was measured during exposure to pictures of urban versus natural environments, rather than after it. Our general expectation was that, on the assumption that nature is effortless to attend to, participants would be more accurate and faster in these tasks when they had natural as opposed to urban images in view. Recognizing target sequences was also expected to be more effortful than recognizing nontarget sequences because more properties needed to be identified in the former type of sequences. This allowed us to explore whether there is a particular level of difficulty at which the nature effect becomes most pronounced.

Indeed, we found that target sequences were recognized significantly slower than nontarget sequences, confirming that the former are indeed more effortful to identify than the latter. However, one of the main findings of Study 3 is that, in contrast to our expectations, participants were faster when sequences – both targets and nontargets – surrounded urban pictures than when they were arranged around natural pictures. This finding *prima facie* speaks against our proposed hypothesis, derived from ART, that the supposed effortlessness of attending to natural versus urban scenes makes that more cognitive resources are available to perform cognitive tasks, eventually resulting in quicker task performance. However, it interlocks with another finding from Study 3, namely that overall, participants were more accurate in recognizing sequences when these were placed around natural versus urban pictures. With nature in view, participants’ responding thus appeared to slow down, but their responses also became more accurate.

It seems, however, that more is going on than a mere speed-accuracy tradeoff. More detailed analyses revealed that the most difficult sequence types, i.e., the target sequences, were more rapidly identified when participants had natural as opposed to urban pictures in view. The reverse pattern appeared for the easier nontarget sequences: these were recognized quicker when they were surrounding urban as opposed to natural images. So, while our original expectation was that nature pictures would lead to overall superior performance (i.e., faster identification times), both for targets and nontargets, we only found a nature advantage for the more cognitive effortful targets.

One interpretation of this last result is that respondents adopted diverging problem solving strategies depending on the type of environmental picture which they saw [48]. When sequences surrounded natural pictures participants might have adopted *systematic* problem solving, during which they recalled all possible properties of the sequences, and systematically checked whether these apply to the sequence they had in view. In contrast, when the sequences were positioned around urban pictures, participants might have engaged more in heuristic rather than systematic problem solving. One possible heuristic for speeding up overall sequence identification could have been to focus only on finding nontargets, which can be quickly identified on the basis of only few properties. Identifying targets could however have suffered from this narrow focus on nontargets, leading to comparatively slower identification times for this type of sequences.

Note that systematic processing might be due to the fact that the environmental stimulus is low on cognitive effort, whereas making recourse to (effortless) heuristics might be a strategy to compensate for the fact that having urban pictures in view commands comparatively more cognitive effort [49]. This pathway is consistent with the suggested effort dimension of fascination, central to ART. We admit however that this interpretation is speculative and further research is required to test whether natural versus urban scenes could respectively lead to more systematic versus heuristic processing, and whether or not this processing style difference is due to a difference in processing effort.

### Limitations

Although the current research addressed some of the shortcomings associated with self-report measures in restorative environments research, we also want to point to a number of limitations of our three studies. A first issue is that restorative experiences typically occur when particular cognitive or emotional resources are missing or low. According to ART, restoration takes place when depleted attentional resources are replenished through exposure to unthreatening natural environments. Therefore, one potential concern with the current studies could be that participants had not been (attentionally) depleted prior to the experiments. So, can our results really contribute anything meaningful to restorative environments research, which heavily focuses on the recovery from depletion?

We have two answers to the previous concern. First of all, research shows that fascination – and its three underlying dimensions – not only drives the restorative effects of contact with nature, but also its so-called “instorative” or vitalizing effects [10]. In other words, the key concepts and constructs central to our three studies also underlie positive nature experiences that are independent from depletion. Thus even without any prior depletion our findings are relevant for the broader field of restorative environments research. A second point is that in ART the three restorative dimensions are seen as prior conditions that make it actually possible for restoration to occur. Therefore, only without prior depletion can we check whether these three dimensions actually characterize fascination with nature and drive restorative experiences.

A second issue relates to the fact that the pictures in our image set differed on a number of important visual dimensions. Specifically, the nature images depicted colorful scenes, whereas those of the urban environments had relatively dull colors. Natural environments also have fractal qualities, which are uncharacteristic to most (modern) urban areas [50]. This raises the question whether some of our findings (e.g., higher levels of positive affect, attentional bias to nature) are not merely an artifact of these visual differences. We think, however, that this question is misguided, because it are exactly qualities like color or fractality which define the difference in (the restorative dimensions of) fascination between urban and natural scenes. Taking away those properties – if possible – would probably substantially wash away the restorative effects of nature. Furthermore, note that in restoration studies it is common to use pictures like the ones in our stimulus set. The field of restoration studies aims to apply its research findings to real life settings (cfr., green interventions), and therefore uses stimulus sets that mirror people’s actual experience of natural versus urban environments – and it is just a fact that those environments differ on qualities like fractality or color.

A third concern relates to the fact that our studies probably provide insight into only a particular aspect of the three suggested dimensions of fascination. For example, while fascination is assumed to have an attentional dimension, it remains somewhat unclear whether this dimension refers to either the process of momentarily *attracting* attention (away from other stimuli), or of *holding* attention for a prolonged period of time, or whether it involves both. It should be clear that in Study 2 we have only measured individual’s attentional bias to the stimuli (i.e., attracting attention), so our findings do not allow us to draw any conclusions about whether natural scenes also hold attention in an enduring way. Similarly, theory on restorative nature experiences says little about whether the effort dimension refers to the low levels of cognitive effort that are required to orient attention towards the natural environment (i.e., automaticity of orienting) or whether it refers to the little effort that is needed to grasp and process the visual information of these environments. While Study 3 could be interpreted as supporting the effort dimension of fascination, the results do not say much about the possible sources of this effortlessness. Note that this last point applies to all three studies. However, in this paper, our main ambition was to explore whether or not the three hypothesized dimensions of fascination actually apply to human encounters with (pictures of) natural environments. We hope that future research will examine, or differentiate, between the potential sources of these dimensions.

### Conclusion

These days, research on the “healing” aspects of natural environments is widely covered by the media and has become a fashionable topic in both the popular and academic press. A decade ago research on restorative nature experiences was mainly published in particular “niche” journals (e.g., *Journal of Environmental Psychology*; *Environment & Behavior*; *Landscape and Urban Planning*), but in the last few years a number of top ranked psychology journals has also provided an outlet for this research [16], [22], [51]. However, despite the increasing academic popularity of the topic of restoration and its wide appeal to the general public, the specific mechanisms(s) underlying nature’s restorative effects are still not well understood. While fascination has been put forward as one of the main proximate causes of restoration, our understanding of how exactly fascination drives restoration has generally been informed by intuition and introspection (i.e., self-reports).

This paper attempted to further uncover the underlying “architecture” of fascination. Specifically, we tried to gather direct and objective evidence to show that when people encounter fascinating natural environments, such environments trigger positive affect, attract their attention, and are relatively effortless to attend to. Two of the three instruments we employed to measure these dimensions – the dot probe paradigm and the affect misattribution procedure – have already proven their validity in experimental psychology research. For the current studies, these instruments proved to be easy to design and implement, and to be suitable for restoration research purposes. Our results with the AMP and DPP *prima facie* confirm that fascinating natural environments more readily trigger positive affect and attract attention than urban environments. We hope that in the future instruments like these will be increasingly implemented in restorative environments research to further test basic assumptions underlying ART and as a complement to introspection-based measurement methods.

The results that were obtained with the third instrument – the cognitively effortful task – were less straightforward to interpret within the ART framework. However, instead of solely seeing this as a weakness, we actually think it can provide an opportunity to revise or refine on particular theoretical assumptions of ART. For example, our findings suggest that the (cognitive) beneficial effects of nature are not general, but apply to a particular range of tasks and task difficulties. Such refinements can have important practical ramifications because in restorative environments research theory and practice are traditionally tightly interlocked [52], [53]. Based on the finding that natural environments and elements can have beneficial psychological and cognitive effects, particular guidelines for urban and landscape planning can be thought out and formulated. As noted in the introduction of this paper, the value of such interventions is underscored by the potentially negative effects of the demands of daily life on our physical and psychological health. Optimal interventions are only possible with sufficient knowledge about the mechanism(s) that underlie restorative nature experiences, and we hope that our paper constituted an attempt to advance that knowledge.
