# Correction: New Methods for Assessing the Fascinating Nature of Nature Experiences

**DOI:** 10.1371/annotation/b4b68a93-1449-4df7-9788-6abe0cbbf6a0

**Published:** 2014-01-22

**Authors:** Yannick Joye, Roos Pals, Linda Steg, Ben Lewis-Evans

The name of the fourth author was listed incorrectly. The correct name is Ben Lewis-Evans. The correct citation is: Joye Y, Pals R, Steg L, Lewis-Evans B (2013) New Methods for Assessing the Fascinating Nature of Nature Experiences. PLoS ONE 8(7): e65332. doi:10.1371/journal.pone.0065332.

